# In search of an evidence-based role for psychiatry

**DOI:** 10.4155/fsoa-2015-0011

**Published:** 2016-02-18

**Authors:** John Read, Olga Runciman, Jacqui Dillon

**Affiliations:** 1 Clinical Psychology, University of East London, Stratford Campus, 1 Salway Road, Stratford, London E15 1NF, UK; 2 Hearing Voices Network, Denmark; 3 Hearing Voices Network, UK; University of East London, UK

**Keywords:** electroconvulsive therapy, evidence based practice, mental health, psychiatric drugs, psychiatry, public opinion

While psychiatrists everywhere are doing their best to help people, their profession is in crisis. Psychiatry is struggling to defend itself from multiple sources of critique, and to reassert its future role. One possibility that is taboo for any profession to consider, however, is that it has little or no useful role. That possibility must be contemplated by others. An evidence based approach to evaluating what good psychiatry contributes to mental health services in the 21st century leads to some challenging conclusions.

Psychiatry's crisis is evidenced in many ways. Most blatant is the international outpouring of criticism at the fifth edition of the Diagnostic and Statistical Manual of Mental Disorders [1], its latest attempt to categorize human distress into discrete psychiatric ‘disorders’. The fact that the attack on the poor science involved was led by the editor of the fourth edition [2], and the Director of the USA's National Institute of Mental Health [3], was embarrassing.

It seems psychiatry is now held in low regard by other medical disciplines. Medical students in numerous countries are uninterested in psychiatry as a career, seeing it as unscientific and ineffective [4]. In one study only 4–7% of UK medical students identified psychiatry as a ‘probable/definite’ career choice, partly because of its poor empirical basis [4]. In a recent survey over 1000 nonpsychiatric medical faculty members, at universities in 15 countries, “did not view psychiatry as an exciting, rapidly expanding, intellectually challenging or evidence-based branch of medicine” ([5], page 24). A total of 90% believed that ‘Most psychiatrists are not good role models for medical students’. The most negative opinions were expressed by neurologists, pediatricians, radiologists and surgeons.

Even more revealing than the survey findings was psychiatry's response to it. The researchers themselves, including a former President of the World Psychiatric Association, wondered whether their colleagues’ opinions are ‘well founded in facts’ or ‘may reflect stigmatizing views toward psychiatry and psychiatrists’. Their own answer to that question becomes abundantly clear when, instead of proposing efforts to address the problems identified by the medical community, such as having little scientific basis, they recommend only ‘enhancing the *perception* of psychiatrists’ so as to ‘improve the *perception* of psychiatry as a career’ [5].

Similarly, all six responses to the survey, in the same edition of the journal, written by 13 psychiatrists (including current and past Presidents of the European Psychiatric Association and the current President of the World Psychiatric Association) dismissed all the concerns raised by the 1057 medical experts and blamed everyone but their own profession, including their supposedly ignorant, prejudiced medical colleagues and the biased media. The titles of these responses included ‘Overcoming stigmatizing attitudes toward psychiatrists and psychiatry’ [6] and ‘Some thoughts on how to improve the image of psychiatry’ [7].

The journal editor did invite one response from outside the profession [8]. To that respondent, and to other commentators, the problem with these senior psychiatrists’ response to criticism seemed obvious:

“While all authors in their own different ways address what might be done to improve psychiatry's image, significantly, not a single psychiatrist thinks to ask what by humanistic standards would appear to be the compulsory question: Insofar as any of the bad image is deserved, exactly how are the ‘patients’ being ill served and what is owed them?” [9]; and
“This strikes me as condescending to the point of arrogance, and, to the extent that it reflects psychiatric attitudes generally, could, in combination with psychiatry's spurious foundations and destructive ‘treatments’, go a long way to explaining the negative perceptions of other medical professions” [10].

One of the six responses [11] did acknowledge some fault on the part of the profession, but only in the past. The 1000 or so medical colleagues are, we are told, behind the times. One wonders, however, whether things are actually getting worse not better. Numerous prominent psychiatrists have recently been exposed engaging in unethical financial dealings with the pharmaceutical industry [12–14].

A discipline claiming a central role should be contributing to three core research domains: conceptualization, causation and treatment. In terms of conceptualization, psychiatry's primary contribution is an ever expanding list of labels [1]. Calling them ‘diagnoses’ cannot disguise the fact that many do not reach minimal scientific reliability levels and have little or no predictive validity for outcome or treatment responsiveness [2,3,15,16]. For example, ‘schizophrenia’ – the flagship of biological psychiatry – requires just two of five symptoms, meaning you can get this ‘diagnosis’ without having anything in common with another person given the same ‘diagnosis’ [15]. Such disjunctive constructs are instantly dismissed as unusable by real scientific disciplines. Even the USA's National Institute of Mental Health, in its unceasing quest for the missing biological causes of human distress, has abandoned the diagnostic approach to classifying mental health problems and acknowledged the need to try to develop some scientifically robust ‘research domains’ [3]. This is not just academic. Labels like ‘schizophrenia’ can, like the biogenetic causal beliefs that tend to accompany them, destroy lives, through prejudice, fear and prognostic pessimism [17–19].

In terms of causation, psychiatry has focused predominantly on chemical imbalances, brain abnormalities and genetics. The failure to provide any findings of substance [15,20,21] does not seem to matter. Merely engaging in this apparently scientific activity seems sufficient to sustain the ‘medical model’. Of course genetics is important but only if we research constructs that exist, using methodology that meets basic standards [21] and only if we acknowledge the role of epigenetic processes whereby genes are activated and deactivated by the environment [22]. The brain's primary role is to respond to the environment but many psychiatrists appear unable to grasp this. Many still do not realize that brain differences between groups can be explained by the effects of childhood trauma on the developing brain [23].

In terms of treatment, research suggests that the safety and efficacy of psychiatric drugs have been grossly exaggerated [12–15,24–30]. For example, the latest best estimates as to the percentage of people who benefit over and above placebo effects are 20% for antipsychotics and even less for antidepressants [24–26]. Furthermore, both antidepressants and antipsychotics have a range of well documented adverse effects, some of which are life threatening [12,13,15,25–27]. A survey of 1829 people on antidepressants found the following rates: sexual difficulties (62%); feeling emotionally numb (60%), withdrawal effects (55%), feeling not like myself (52%), agitation (47%); reduction in positive feelings (42%), caring less about others (39%) and suicidality (39%) [27]. Despite clear evidence that antipsychotics can cause brain degeneration [25,28,29] and shorten life span [25,29,30], we still grant psychiatry, via mental health legislation, the right to force people to take them against their will. Drugs giant Otsuka has just applied to the US FDA to be able to insert a chip in Abilify so as to monitor ‘medication compliance’ [31].

Electroconvulsive treatment, which is undergoing a renaissance in countries most strongly dominated by biogenetic ideology, such as the USA and Australia, has no lasting benefit at all compared with placebo [32]. Unsurprisingly, it can cause long lasting or permanent cognitive dysfunction, primarily in the form of anterograde and retrograde amnesia [32].

Meanwhile, perhaps the most exciting development in the field of ‘treatment’ is the hearing voices movement. While the evidence base for the efficacy of the hearing voices groups being run by voice hearers in 35 countries is in its infancy [33–35], the groups do not cause stigma, pessimism, diabetes, brain damage, suicide or shortened life span.

Despite all this, biological psychiatry is trying to expand the reach of what others consider to be an unscientific, reductionist, simplistic and pessimistic ‘medical model’. Some psychiatrists have bemoaned what they call the poor ‘mental health literacy’ (i.e., one's willingness to agree with biological psychiatrists about the causes of human distress), not only of people in their own countries and cultures, but of people in numerous ‘developing’ countries, including India, Pakistan, Bali, Nigeria and Malawi [18]. Terms like ‘misinformation’ and ‘lack of knowledge’ are used to describe spiritual and social causal beliefs. A typical conclusion is ‘Interventions aimed at increasing the mental health literacy of traditional healers are essential’ [36]. These researchers consistently express concern that other cultures’ beliefs limit the use of psychiatric drugs.

There is even an international organization, ‘Global Mental Health’, designed to bring the supposed superiority of the Western approach to the rest of the world [37]. There seems to be no understanding that supplanting indigenous beliefs with those of a dominant culture is a cornerstone of colonization. Also conveniently ignored are the findings of WHO studies that recovery rates for ‘schizophrenia’ are significantly higher in ‘developing’ countries than in ‘developed’ countries [16,38]. Meanwhile, within the USA, researchers are alarmed that African–Americans insist on believing in ‘debunked theories of schizophrenia that focused on the family's effect on causing schizophrenia’ [39]; and bemoan the poor ‘psychosis literacy’ of Latinos, especially their failure to make ‘illness attributions’ and their insistence that the ‘social world’ is important in understanding psychosis [40].

What role, if any, should be played by a profession whose research and thinking are so heavily influenced by drug companies [12–14] and which has produced so little of benefit to service users for 50 years? One potential role would be the traditional doctors’ function of attending to real medical illnesses. But even the official journal of the World Psychiatric Association has bemoaned the ‘suboptimal medical care’ provided by psychiatry [41], not to mention that some of service users’ most serious health conditions are caused by psychiatric drugs.

Despite their relative inefficacy and dangerousness, psychiatric medications can be helpful (as a last resort, and for a short period). Therefore, mental health teams do need access to people with prescribing rights. So there is a useful role for psychiatrists, but only if they take an evidence-based approach, which concedes that a range of more effective and safer treatments should be offered first, that adverse effects should be fully disclosed and that no medical treatment should be forced on anyone against their will. In other medical specialties forcing a patient to receive a treatment constitutes ethical misconduct and is severely punished.

Finally, we should remember that the public, in other words users of mental health services, have a strong preference for psycho-social explanations and treatments. In 24 of 25 countries where surveys have been conducted the public believes that social factors play a much greater role than genes or chemical imbalances in the etiology of mental health problems, with the only exception being the USA [42,43]. Similarly, in 14 out of 15 countries the public prefers talking therapies and social support to drugs or electroshock [42]. The evidence summarized above suggests that they may be right.

But is anyone paying attention to the public or the research? Currently, a lack of nonmedical staff – including peer support workers – especially in positions of leadership, is limiting the implementation and availability of nonpharmacological interventions. What would happen if managers, funders and politicians really took all this research evidence and public opinion into consideration when deciding what sort of services to provide and what sort of staff to employ?
